# School- and Leisure Time Factors Are Associated With Sitting Time of German and Irish Children and Adolescents During School: Results of a DEDIPAC Feasibility Study

**DOI:** 10.3389/fspor.2020.00093

**Published:** 2020-07-23

**Authors:** Johanna Sophie Lubasch, Barbara Thumann, Jens Bucksch, Lara Kim Brackmann, Norman Wirsik, Alan Donnelly, Grainne Hayes, Katharina Nimptsch, Astrid Steinbrecher, Tobias Pischon, Johannes Brug, Wolfgang Ahrens, Antje Hebestreit

**Affiliations:** ^1^Leibniz Institute for Prevention Research and Epidemiology—BIPS, Bremen, Germany; ^2^Organizational Health Services Research, Department for Health Services Research, Faculty IV School of Medicine and Health Sciences, Carl von Ossietzky University Oldenburg, Oldenburg, Germany; ^3^Faculty III—Prevention and Health Promotion, Heidelberg University of Education, Heidelberg, Germany; ^4^Health Research Institute, University of Limerick, Limerick, Ireland; ^5^Max Delbrück Center for Molecular Medicine, Berlin, Germany; ^6^Amsterdam School of Communication Research (ASCoR), University of Amsterdam, VU University Medical Center, Amsterdam, Netherlands; ^7^Faculty of Mathematics/Computer Science, University of Bremen, Bremen, Germany

**Keywords:** sedentary behavior, contextual factors, cross-contextual factors, multi-level models, accelerometry

## Abstract

**Objective:** The study aims to investigate to what extent school- and leisure time-related factors are associated with sedentary behavior during school in German and Irish children and adolescents.

**Methods:** The study based on a sample of 198 children and adolescents surveyed in 2015. Sedentary and activity behavior were measured using the activPAL physical activity monitor. Information on socio-economic status, school- and leisure-time related factors were provided by questionnaires. Associations between school- and leisure time-related factors and sedentary time during school were estimated using linear multi-level models.

**Results:** Access to play equipment in school was associated with reduced sitting time (hours/day) of children (ß = 0.78; 95%CI = 0.06–1.48). Media devices in bedroom and assessing the neighborhood as activity friendly was associated with increased sitting time of children (ß = 0.92; 95%CI = 0.12–1.72 and ß = 0.30; 95%CI = 0.01–0.60, respectively). The permission to use media devices during breaks was associated with increased sitting time (hours/day) of adolescents (ß = 0.37; 95% CI = 0.06–0.69). A less safe traffic surrounding at school was associated with reduced sitting time of adolescents (ß = −0.42; 95% CI = −0.80 to −0.03).

**Conclusion:** Results suggest that school- and leisure time-related factors are associated to the sedentary behavior during school. We suggest that future strategies to reduce sedentary time should consider both contexts.

## Introduction

Sedentary behavior (SB) comprises all waking behaviors in a sitting or reclining posture with a low energy expenditure (Tremblay et al., 2017). SB is the collective term used for the levels and patterns of sedentary time (ST) (Tremblay et al., 2017). It is associated with various health risks (Hamilton et al., 2008; Owen et al., 2010). In many societies changes in transportation, work-place structure, and entertainment-technologies have led to an increased amount of time spent in sedentary pursuits. This increase of sedentary behavior holds various health risks whereby investigations on the amount of time spent sedentary came into the focus of public health research (Hamilton et al., 2008; Owen et al., 2010). Although the strength of the association between SB and health outcomes in childhood and adolescence is not yet conclusively researched (Chinapaw et al., 2015; Suchert et al., 2015) there is evidence to suggest that levels of sedentary behavior during adulthood are established from childhood (Biddle et al., 2010). This concludes that reducing ST is an appropriate and thus important intervention strategy for children and adolescents to develop and adopt a healthy lifestyle. Different studies showed that European children and adolescents spend between 345 and 578 min per day sedentary (Ruiz et al., 2011; Ekelund et al., 2012; Gracia-Marco et al., 2013; Chinapaw et al., 2014; Husu et al., 2016) and that children and adolescents spend about 50% of their leisure time and 70% of their school time sedentary (Mantjes et al., 2012; Arundell et al., 2016a; Beck et al., 2016). During school as well as during leisure time higher levels of ST were particularly observed in girls compared to boys and higher age was linked to more sedentary activities (Pate et al., 2011; Ruiz et al., 2011; Ekelund et al., 2012; Mantjes et al., 2012; Gracia-Marco et al., 2013; Saunders et al., 2013; Stierlin et al., 2015; Arundell et al., 2016b). Moreover, in general higher levels of SB were reported for children from lower socio economic status (SES) background than from higher SES background (Pate et al., 2011).

The health behavior of children and adolescents has been found to be associated with multiple factors of different contexts which co-occur and interact with each other (National Research Council, 2004), and those factors vary with advancing age (Halfon and Hochstein, 2002). The school and the leisure time context provide different factors for SB of children and adolescents. Different studies highlight that school related factors of SB include the school surrounding (Mantjes et al., 2012; Stierlin et al., 2015), the school's internal structures (for example length of breaks, rules regarding media consumption) (Mantjes et al., 2012; Morgan et al., 2016; Morton et al., 2016) and the school's equipment (Mantjes et al., 2012; Ridgers et al., 2013; Stierlin et al., 2015; Morton et al., 2016). Factors of the leisure time context associated with SB are related to the home environment (Pate et al., 2011; Maitland et al., 2013; Kaushal and Rhodes, 2014; Draper et al., 2015), the home surrounding and in the neighborhood (Maitland et al., 2013; Kaushal and Rhodes, 2014). School-aged children spend up to 30 h and more per week in school and around school buildings (Sekretariat der Ständigen Konferenz der Kultusminister der Länder in der Bundesrepublik Deutschland., 2013; Department of Education and Science Primary Branch, 2020). With 9–10 h of sleep per 24 h (Iglowstein et al., 2003) children and adolescents spend about 31% of their waking day at school. Therefore, the current study assessed the role of SB within the school context (all activities during school hours) and within the leisure time context (time out of school) for children and adolescents independently. Activities during school hours were differentiated into active (e.g., physical education, activity during breaks) and sedentary (e.g., sitting during lessons) time. Little is known about whether and how the contextual factors influence the SB of children and adolescents across specific contexts. However, family environments may have an important influence on SB. It is widely accepted sedentary behaviors adopted by parents can impact the sedentary behaviors of their children (Granich et al., 2010; Jago et al., 2010; Muñoz-Galiano et al., 2020). Consequently, the present analysis considered the role of SB within the home environment in conjunction with the role of SB within the school environment. Therefore, the aim of this study was to investigate whether the SB of children and adolescents during school time is only influenced by basic (age, SES) and school-related factors, or whether leisure time-related factors also influence SB.

## Methods

### Study Participants

**Figure 1:**
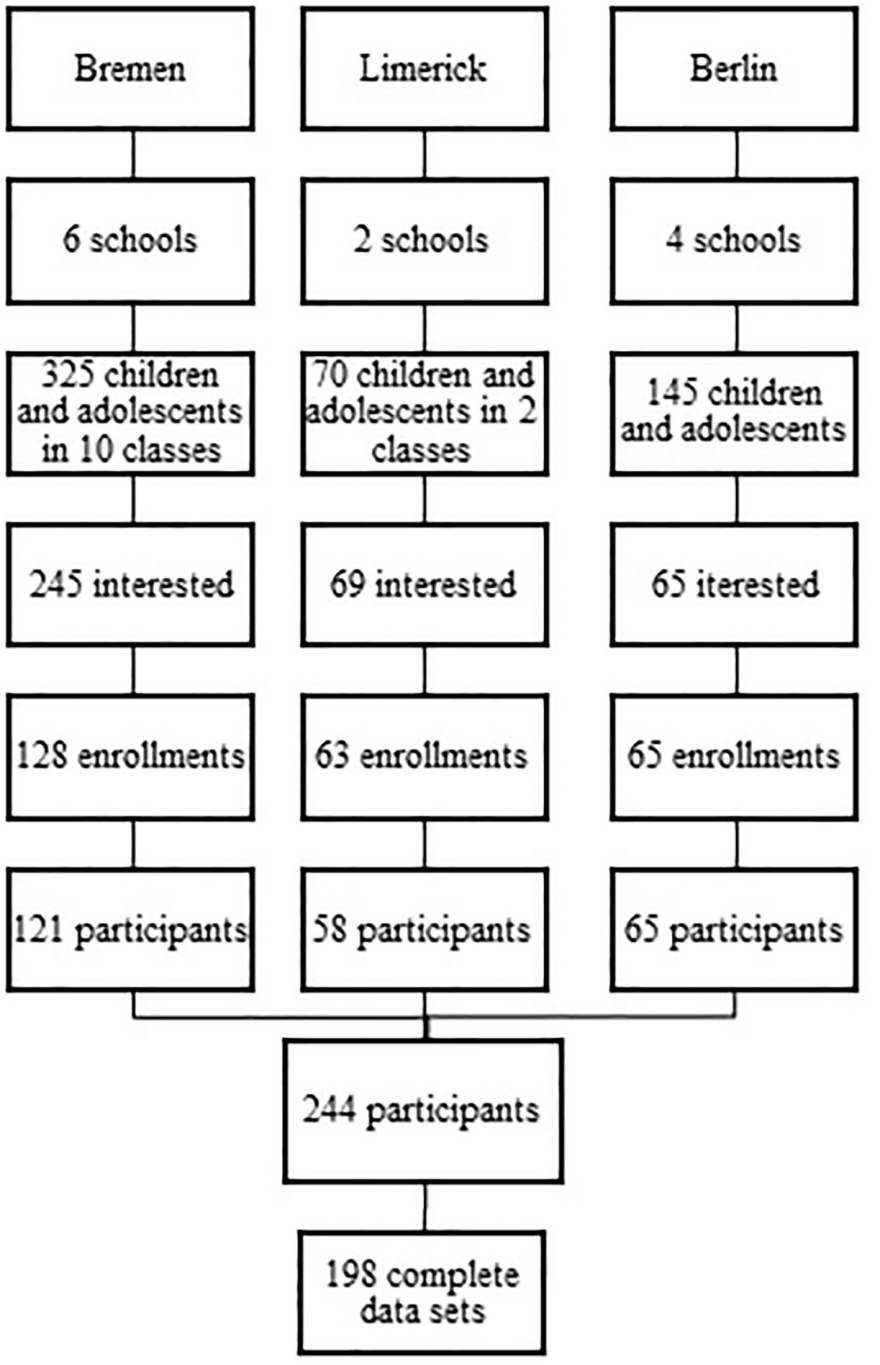
Recruitment of the study population.

In 2015, data for the present study were collected by means of a feasibility study that aimed to investigate a newly developed instrument designed to measure SB and media use in children and adolescents for the purpose of pan-EU surveys within the Determinants of Diet and PA Knowledge Hub (DEDIPAC KH) project (Lakerveld et al., 2014, 2017). Data assessment took place in Bremen: May—December 2015, in Berlin: February—May 2016 and in Ireland; February—March 2016. Children and adolescents were enrolled via schools; the enrollment of siblings was not envisaged. For this investigation children (7–8 years) and adolescents (14–15 years) who lived with their families were invited to participate together with the person having the care and custody of the child. The age-groups were chosen with the aim to survey two groups of participants in two school-contexts (primary vs. secondary school) who have presumably different SBs. A similar number of girls and boys in the sample was pursued. Each participating center obtained ethical approval from the local responsible authorities in accordance with the ethical standards laid down in the 1964 Declaration of Helsinki and its later amendments. Parents and children above 10 years of age provided written informed consent. Children and adolescents gave oral consent for wearing the activPAL device and adolescents gave also consent for completing the questionnaire. It was explicitly pointed out that the participation is voluntarily and that the consent may be withdrawn at any time without notice of any reason and without incurring disadvantages. The process of the recruitment of the study population is shown in Figure 1.

### Questionnaire Data

Questionnaires were developed in English, and—for use in Germany—translated into German, and then translated back into English. Data on SB during school and during leisure time as well as the potential school- and leisure-time factors have been collected on seven consecutive days. Adolescents self-reported these data and parents of primary school children proxy-reported for their children. Information on socio-economic status was reported by parents in both children and adolescents. General information on the school rules, break times and school surroundings was obtained via questionnaire filled-in by the principal or teacher. With regard to the questionnaire data, it must be acknowledged that as the primary purpose of the newly developed instrument was to assess SB in survey, the questions included with the survey were designed to be as short as possible and therefore may have lacked precision.

#### Socio-Demographic Factors

Sex and age were self-reported. Participants aged <10 years were categorized as children and participants aged more than or equal to 10 years as adolescents. As this feasibility study was undertaken for the purpose of surveys within the DEDIPAC, the questionnaires were kept as short as possible. The highest educational level of both parents was used as the indicator for the SES of the included families. This was included as it is deemed the strongest socio-economic predictor of health or health behaviors in different contexts (Winkleby et al., 1992; Härkönen et al., 2018) including physical activity and SB (Loyen et al., 2016; Muñoz-Galiano et al., 2020), highest educational level of both parents was classified by the International Standard Classification of Education (ISCED) 2011 (United Nations Educational, Scientific and Cultural Organization, 2016). In accordance with previous studies (Hebestreit et al., 2016), ISCED levels zero to two have been defined as low, levels three and four as middle and levels five to eight as high SES (variable labels: low = 1, middle/high = 0).

#### School-Related Factors

Traffic safety around the school was assessed by items concerning traffic volume, availability of sidewalks, traffic lights and crosswalks, speed of cars driving past the school, road safety and the possibility to reach the school by foot, each assessed using a 5-point Likert-scale. The answers were coded with higher values indicating higher traffic safety. The question about traffic volume was therefore recoded so that higher volume received a lower score indicating less safety. The average value of all six statements was calculated per participant and higher traffic safety (mean value ≥ 2.5) was coded as “0” and lower traffic safety (mean value <2.5) was coded as “1.”

Two questions concerning the permission to use a computer and to watch TV or videos during breaks have been categorized and summarized as one dichotomous variable (“0” = “no permission to use media devices”; “1” = “sometimes or always permitted to use media devices”).

For each day and participant, the respective number of breaks between school lessons longer than 15 min was calculated by taking the beginning and the duration of breaks at school and of the participants stay at school into account. A 15 min threshold was chosen as Mantjes et al. (2012) have identified that breaks longer than 15 min result in more favorable outcomes regarding sedentary behavior compared to shorter breaks.

To collect data on extracurricular sports programs, adolescents and parents of children where asked about their daily schedule of the previous week.

Teachers and principals were asked, whether children and adolescents were “always,” “sometimes,” or “never” allowed to use the play equipment of the school and to play ball games during breaks. The answers were collated into one dichotomous variable (“0” = “always access to play equipment”; “1” = “sometimes or never access to play equipment”).

#### Leisure Time-Related Factors

Characteristics of the residential neighborhood surroundings were assessed by items concerning availability of parks and playgrounds, traffic and crime security, possibilities to play outside and active transportation with a 5-point Likert-scale. The answers have been coded with higher values indicating higher likelihood of activity supporting lifestyles. The mean value of all four statements per participant was calculated and coded into “0” = highly (average value > 3.5), “1” = middle (mean value 2.5— ≤ 3.5) and “2” = less (mean value <2.5) activity friendly neighborhood.

From the number of TVs and computers available in the bedroom of the participants a dichotomous variable has been formed (“0” = “own media device in bedroom”; “1” = “no own media device in bedroom”). Information on rules concerning watching TV, using an electronic device for pleasure at home were summarized into one dichotomous variable (“0” = “rules concerning media use”; “1” = “no rules concerning media use”). The media consumption behavior of the parents was assessed by items about the hours they spent watching TV or using a computer per day (never, <1, 1–2, 2–3, 3–4, and more than 4 h/d). A scale was calculated measuring the average extent of media use per day per parent was calculated (consumption behavior of week days weighted five times summarized by consumption behavior of weekend days weighted two times and the divided by seven).

The frequency of encouragement to pursue a non-sedentary activity through family members or friends (never, <1 ×/week, 1–2 ×/week, 3–4 ×/week, more than 4 ×/week) was dichotomised into one variable (“0” = “participant is motivated at least sometimes”; “1” = “participant is not motivated or only rarely motivated”).

### Accelerometry

SB was measured objectively using the activPAL 3 micro PA monitor (PAL Technologies Ltd., Glasgow, United Kingdom), which is a small lightweight device that was attached to the midline anterior aspect of the upper thigh. Children and adolescents were asked to wear the devices for 9 days and preferably to only remove them in case of imminent prolonged immersion into water (e.g., swimming or bathing). To access the recorded data the activPAL proprietary software (activPALTM Professional V5.9.1.1) was used. Data for the entire week of recording were exported to Microsoft Excel (Microsoft Corporation, Microsoft Excel 2010. One Microsoft Way, Redmond, WA, USA). All activPAL data were recorded and analyzed in accordance with the measurement of sedentary patterns and behaviors as outlined by Dowd et al. (2012). Briefly, the number of seconds the participants spent sedentary/lying, standing and stepping for each 15 s epoch were displayed. Consistent with previous literature, each participant was required to provide at least 3 weekdays and 1 week-end day of activPAL data (Dowd et al., 2012). Non-wear time was defined as a minimum of 60 min of consecutive zero accelerometer counts during waking hours. A MATLAB® (The MathsWorks Inc., MA, USA) custom software program was used to examine the sedentary function of the activPAL device A sedentary epoch was defined as an epoch spent entirely in sedentary activities (sitting/lying) and a non-sedentary epoch was defined as an epoch with <15 s of sedentary activity. Each 15 second epoch was coded as either being a sedentary (code = 1) or a non-sedentary (code = 0) epoch. The MATLAB software then sequentially examined each daily activPAL file, and for each consecutive sedentary bout it identified the start and end of the sedentary bout and the amount of time spent in each consecutive bout.

To consider only the waking hours, bed hours were identified and were subtracted from total daily STs. To obtain data on ST during school hours and during leisure time, children self-reported the time they started and finished each school day. Data on daily school times (beginning/end of school) was then used to separate STs during school time from STs during leisure time. For the purpose of the present analysis ST was calculated in hours per day and PA in minutes per day.

Due to incomplete questionnaire or accelerometer data, implausible daily STs or missing data on school hours 46 participants were excluded from the analysis. In the end 198 out of 244 participants provided complete and plausible information (questionnaire and accelerometer) with a total of 1,094 measured days for the analysis.

### Statistical Analysis

To investigate the association between context-independent and school- and leisure time-related factors with total daily SB during school hours multi-level linear regression analysis was used with random intercepts to control for cluster effects. Table S1 provides further information on the independent variables. Multilevel analysis was chosen to control for dependence of observations within one individuum (Hox, 2010). The first level was formed by daily repeated measurements and the second level by the individuals. A possible third level in terms of schools was not introduced as it only contains 13 cases. Table S2 provides an overview on the measurement levels of each variable. The analysis was conducted separately for children and adolescents. For each age group one model without exposure variables, a second model with the socio-demographic variables, a third model with the school- and leisure time factors and a fourth model with all variables was set up. The Akaike information criterion (AIC) was used to identify the model which was neither too complex nor too simple (Guthery, 2008). A difference of the AIC of two models of a minimum ten allowed no empirical support for the model with the higher AIC (Burnham and Anderson, 2010). The analysis was conducted with IBM SPSS Statistics (IBM, NY, USA) version 24 with a level of significance of 0.05.

## Results

### Descriptive Results

In total 1,094 days of measurement were available for the analysis. The frequencies and distribution of the exposure variables and ST during school hours (outcome) in the study population can be found in Table 1.

**Table 1:** Characteristics of the study population.

			**Age [12.3 (3.3)] years (SD)**		
			**Total** **(*n* = 198)**	**Children** **[7.4 (0.5) years (SD), *n* = 62]**	**Adolescents** **[14.4 (0.7) years (SD), *n* = 136]**
	Sedentary time during school	Hours [mean (SD) |%^a^]	4.0 (1.0) | 63.4%	3.5 (0.8) | 50.6%	4.3 (1) | 69%
	SES	High [*n* (%)]	112 (64.7%)	42 (70.0%)	70 (61.9%)
School-related factors	MVPA^b^ during school	Minutes (mean (SD) |%^a^)	13.5 (11) | 3.4%	17 (11.9) | 4%	11.9 (10.2) | 3.1%
	Traffic safety around school	High [*n* (%)]	91 (46.0%)	32 (51.6%)	59 (43.4%)
	Number of daily breaks longer than 15 min	Number [mean (SD)]	1.9 (0.5)	2.3 (0.6)	1.8 (0.4)
		1 [*n* (%)]	50 (25.3%)	22 (35.5%)	28 (20.6%)
		2 [*n* (%)]	72 (36.4%)	0 (00.0%)	72 (52.9%)
		3 [*n* (%)]	76 (38.4%)	40 (64.5%)	36 (26.5%)
	Allowed to use media devices	No [*n* (%)]	151 (76.3%)	62 (100%)	89 (65.4%)
	Participation in extracurricular sports program	Hours [mean (SD)]	0.1 (0.4)	0.2 (0.4)	0.1 (0.4)
		Yes [*n* (%)]	39 (19.7%)	20 (32.3%)	19 (14.0%)
	Availability of play equipment	Unrestricted [*n* (%)]	79 (39.9%)	27 (43.5%)	52 (38.2%)
Leisure time-related factors	MVPA^b^ during leisure time	Minutes [mean (SD) |%^b^]	22.8 (20.1) | 3.9%	17.3 (14.7) | 3.5%	25.2 (21.7) | 4.1%
	Neighborhood quality	High [*n* (%)]	70 (59.8%)	24 (40.0%)	46 (80.7%)
		Middle [*n* (%)]	26 (22.2%)	20 (33.3%)	6 (10.5%)
		Low [*n* (%)]	21 (17.9%)	16 (26.7%)	5 (08.8%)
	Presence media device in bedroom	No [*n* (%)]	87 (43.9%)	55 (88.7%)	32 (23.5%)
	Rules concerning media use at home	Yes [*n* (%)]	95 (50.0%)	40 (71.4%)	55 (41.0%)
	Daily media consumption per parent	Score [mean (SD)]	2.8 (0.7)	2.7 (0.7)	2.9 (0.8)
	Encouragement for non-sedentary activity	At least sometimes [*n* (%)]	81 (42.6%)	37 (66.1%)	44 (32.8%)

a of school hours;

b *MVPA, Moderate-Vigorous Physical Activity*.

At the time of the survey, the children were on average 7.4 (0.5) years [standard derivation (SD)] and the adolescents 14.4 (0.7) years (SD) old. The proportion of adolescents in this study population was higher (68.7%, *n* = 136) than the proportion of children. More than half of the participants were female (55.6%) and from families with high SES background (64.7%). On average, participants were sedentary for ~4 h (63.4%) during school. Children spent less time sedentary and were more active than adolescents, in both absolute and relative measures.

### Results of the Multi-Level Analysis

The AIC of the models became smaller when more exposure variables were added to the model, indicating that the model fit increases with the addition of factors to the model (see Table 2). The regression analysis showed that those children, who had restricted or no access to play equipment in school, reported a longer ST (hours per day) (ß = 0.78; 95% confidence interval (CI) = 0.06−1.48) during school compared to children with unrestricted access to play equipment (Table 3). Moreover, it was observed that those children living in neighborhoods that were perceived to be less activity friendly had a shorter ST (ß = −0.30; 95%CI = −0.60 to −0.01) compared to children who lived in neighborhoods perceived to be highly activity friendly. It was further observed that children with media devices in their own bedrooms reported a longer ST during school (ß = 0.92; 95%CI = 0.12–1.72) than children without a media device in their bedroom. Moreover, results revealed that the absence of rules concerning media use in school related to longer ST and a school surrounding evaluated to be less safe with less ST of adolescents during school (ß = 0.37; 95%CI = 0.06–0.69 and ß = −0.42; 95%CI = −0.80 to −0.03, respectively).

**Table 2:** AIC and AIC difference.

**Information criterion**		**Model 1^**a**^**	**Model 2^**b**^**	**Model 3^**c**^**	**Model 4^**d**^**
Children	AIC	578.42	464.69	221.52	179.34
	AIC difference		−113.74	−243.17	−42.18
Adolescents	AIC	1573.34	907.49	792.13	252.22
	AIC difference		−665.85	−115.36	−539.90

a without exposure variables;

b sex, age and SES as exposure variables;

c school-related variables added to model 2;

d *leisure time-related variables added to model 3*.

**Table 3:** Results of multilevel models on the association between school and leisure time related factors and sedentary behavior during school (measured in hours/day).

		**β** **(95% CI**^****b****^**;** ***p*****-value)**	
**Parameter**		**Children**	**Adolescents**
Basic variables	Time spent in school (hours/day)	**0.44 (0.14/0.74; 0.02)**	**0.71 (0.60/0.82; 0.00)**
	SES middle/low (ref. high)	−0.45 (−1.20/0.31; 0.32)	−0.08 (−0.40/0.23; 0.66)
	Sex female (ref. male)	0.20 (−0.18/0.59; 0.38)	0.27 (−0.01/0.55; 0.11)
School–related variables	MVPA^c^ during school (minutes/day)	**−0.02 (−0.03/−0.01; 0.01)**	**−0.04 (−0.05/−0.04; 0.00)**
	Low traffic safety around school (ref. middle and low)	−0.23 (−0.80/0.34; 0.51)	**−0.42 (−0.80/−0.03; 0.04)**
	Breaks longer than 15 minutes (number)	0.34 (−0.23/0.90; 0.32)	0.12 (−0.23/0.47; 0.57)
	Access to media devices in school (ref. no/limited access to media devices)	/^a^	**0.37 (0.06/0.67; 0.03)**
	Participation in extracurricular sports program (number of daily lessons)	0.35 (−0.6/0.75; 0.16)	−0.15 (−0.35/0.05; 0.22)
	No/limited access to play equipment (ref. unrestricted access)	**0.77 (0.06/1.48; 0.04)**	−0.10 (−0.40/0.21; 0.60)
Leisure time–related variables	MVPA^c^ during leisure time (minutes/day)	−0.00 (−0.01/0.01; 0.93)	−0.00 (−0.01/0.00; 0.60)
	Less activity friendly neighborhood (ref. activity friendly neighborhood)	**−0.30 (−0.60/−0.01; 0.01)**	−0.21 (−0.44/0.01; 0,12)
	Media devices in own bedroom (ref. no media devices in own bedroom)	**0.92 (0.12/1.72; 0.04)**	0.13 (−0.15/0.42; 0.44)
	No rules concerning media use at home (ref. rules concerning media use at home)	0.33 (−0.20/0.87; 0.30)	−0.18 (−0.44/0.08; 0.25)
	Media consumption parents (scale 1–6)	0.33 (−0.03/0.69; 0.13)	0.15 (−0.01/0.31; 0.13)
	No encouragement for non-sedentary activity (ref. encouragement for non-sedentarism)	0.05 (−0.33/0.042; 0.84)	0.12 (−0.19/0.43; 0.52)
	Sedentary time during leisure time (hours/day)	0.11 (−0.08/0.31; 0.34)	−0.05 (−0.14/0.03; 0.31)
	Time spent out of school (hours/day)	−0.12 (−0.34/0.09; 0.34)	0.02 (−0.09/0.13; 0.80)

**Significant on 0.5-level**;

a not able to be calculated due to low percentage of children being allowed to use media devices in school;

b Confidence Interval;

c *MVPA, Moderate-Vigorous Physical Activity*.

## Discussion

This feasibility study assessed the association between school- and leisure time related factors and SB of children and adolescents during school hours. For children we observed that both the school-and leisure time level factors play a role in the accumulation of ST during school hours. For adolescents we only observed that school-related factors are associated to the SB during school.

The SB of children and adolescents in this study is comparable to other studies. Mantjes et al. (2012) observed that a proportion of 69% was spent sedentary during school which is comparable to the observed 63.4% of school time spent being sedentary in our study. Our results also show, that children spent less time being sedentary during school hours that adolescents did. This supplements previous results showing that children spend less time being sedentary during leisure time than adolescents (Arundell et al., 2016a). This leads to the assumption that children make more use of opportunities to be active during breaks or classes than adolescents.

Our data suggests that there is an association between rules concerning media use in school and total ST during school hours in adolescents; this is in line with previous results from Pate et al. (2011) who reported that the restriction of media use at home was associated with lower levels of SB among children and adolescents. This finding adds important information to current knowledge and may inform the development of intervention studies and policies aiming to reduce ST in this age group. The results suggest that interventions promoting a reduction of ST during school hours may be most successful when implementing rules on media use during school time. In the leisure time context many interventions were successful in reducing ST by reducing media use, thus these concepts might also serve school interventions as a means to reduce ST. An example of a successful measure is to place a time limit on media consumption (Minges et al., 2015). Moreover, the encouragement of a TV-free week showed promising results (Altenburg et al., 2016). Subsequently rules might be an important measure to reduce the adolescents' media use and therewith to reduce SB during school. Still, as reported earlier (Hebestreit et al., 2010) the clear communication of rules is key.

The association between access to play equipment during school and reduced SB in children and adolescents (Mantjes et al., 2012; Ridgers et al., 2013; Morton et al., 2016) was only observed for children. A possible explanation for this might be that adolescents lose interest in play equipment. It further points out, that interventions are needed to reduce ST during school, especially for adolescents. Thereby, one approach is the integration of standing desks within the classroom environment (Altenburg et al., 2016). This on the one hand has the potential to reduce ST and on the other hand increases standing time (Minges et al., 2016). Further measures to reduce ST during school are activity breaks during class time at school, the performance of active lessons and the assignment of active homework (Salmon, 2010).

In contrast to previous findings (Mantjes et al., 2012; Stierlin et al., 2015) a school surrounding evaluated as safe was associated with higher ST in adolescents. However, we assume that traffic safety is more relevant for SB after school and not during school, because during school adolescents are on the school grounds itself. The association might be mediated by other factors which were not included in the model. Moreover, one has to consider that the school surrounding was assessed by teachers or principals and not objectively which might heave lead to distortions.

Concerning cross-context effects, it was observed that the perception of the activity friendliness of the neighborhood had an effect in unexpected direction on the sedentary behavior in school. One explanation might be that the assessment of the neighborhood to be activity friendly is an indicator of higher family SES or higher educational levels of the parents. The result could be that parents with higher educational levels (living in presumably more activity friendly neighborhoods) place more value on educational aspects (doing homework) which might lead to more ST at home as was initially observed (Muñoz-Galiano et al., 2020). Previous studies reported an association between parental education level and achievement-oriented behavior (e.g., high reading frequency) of the child. Hence, those children—even though having more opportunities to be active in the neighborhoods—may spend more time sedentary at home (doing homework, playing an instrument) and also during school (learning, exercises) as they are most likely used to concentrate (and sit) in longer bouts. Also, those children are less likely to encounter behavioral problems such as early aggression, that hampers good relationships with teachers but also the children's academic and intellectual development over time (Dubow et al., 2009). However this suggestions can neither be strengthened by results of our study (although lower SES is related to more SB this association is not statistically significant) nor by previous findings of the literature (Pate et al., 2011). We therefore further suggest that the effect does not describe a causal relationship and that the neighborhood quality is not relevant for the sedentary behavior of children and adolescents during school. Furthermore, the subjective assessment of the activity friendliness of the neighborhood might lead to distortions due to that fact that the subjective assessments or are not comparable to each other and might be under- as well as overestimated. The estimations of other cross-context effects on SB during school showed that leisure-time related parameters which have been assumed to context-internally decrease the ST also might decrease ST during school. Although only few of the effects are statistically significant it is assumed that cross-contextual factors are important for the SB of children and adolescents. This assumption is supported by the fact that our statistical model containing the cross-context variables has decreased the AIC. Thus, cross-context variables contribute to a higher variance explanation in the regression model. Consequently, we assume that cross-context variables play an important role explaining the development of high STs. To our knowledge, until now no other study examined this influence of cross-contextual factors on SB during school hours. Subsequently, further studies are needed to better understand the effect of cross-context variables on the SB of children and adolescents during school time.

### Strength and Limitations

The present study provides data of high quality and precision in terms of objectively measured PA and SB. Since the data assessment was undertaken across seasons which accounted for seasonal fluctuations in SB. However we have to keep in mind that the primary purpose of the study was to investigate the feasibility of newly developed instruments (Lakerveld et al., 2014, 2017), and not purposefully to address specific questions on determinants of SB. Therefore, the sample size presented here was not powered to address the research question, and the cross-sectional design means that relationships detected may not be causative. Although some of the variables have been conducted on the school level and have been the same for every child or adolescent of one school, this level could not be considered in the analysis. Thereby, the multi-level analysis might have been distorted because for the variables conducted on the school level the assumption of independent observations of children and adolescents from one school was violated (Hox, 2010). Moreover, the self-reporting of domain-specific indicators such as the neighborhood surrounding and the traffic safety around school might have been subject to misreporting due to recall bias or social desirability bias (Adams et al., 2005). The perception of for example security and traffic volume might differ between individuals. Still, the combination of self-reported information on domains (Sprengeler et al., 2017) with objectively measured data should be considered to receive most accurate estimations on where ST amounts most during the day.

## Conclusion

The results of this study partly confirm findings from recent studies concerning the associations of different factors with the SB of children and adolescents. It is suggested that leisure-time related parameters are associated with the SB of children during school. It was shown that some of the associations vary concerning the strength and the direction of the estimator between children and adolescents. Further studies are necessary to verify and to compliment findings from this study. Finally, we believe that detected associations did not reach the significance threshold only because of the small sample and still give important information for public health stakeholders, policy makers, and researchers.

Future interventions improving the SB of children and adolescents should be carried out across all contexts for children and adolescents. In this study it was observed that increasing and decreasing factors are associated with the SB cross-contextual, so the improvement of the SB in one context alone might not be sufficient. It might be beneficial to combine successful approaches in both the school and the home environment. Moreover, interventions should be individualized for different target groups.

## Data Availability Statement

The datasets generated for this study are available on request to the corresponding author.

## Ethics Statement

Each participating center obtained ethical approval from the local responsible authorities in accordance with the ethical standards laid down in the 1964 Declaration of Helsinki and its later amendments.

## Author Contributions

JL together with AH and JBu drafted the first version of the manuscript. GH, NW, AD, and JL were involved in the procession and analysis of accelerometer data. All authors provided feedback, contributed to further versions, read and approved the final manuscript, and were closely involved in the data collection and provided to the interpretation of results.

## Conflict of Interest

The authors declare that the research was conducted in the absence of any commercial or financial relationships that could be construed as a potential conflict of interest.
