# Supplementary material for: School- and Leisure Time Factors Are Associated With Sitting Time of German and Irish Children and Adolescents During School: Results of a DEDIPAC Feasibility Study
Source: Front Sports Act Living. 2020 Jul 23;2:93. doi: 10.3389/fspor.2020.00093 (PMC7739729; doi:10.3389/fspor.2020.00093)
Supplement: Supplementary file 1 [file Data_Sheet_1.docx]

Table S1: Characteristics of independent variables

| Variable | Previous literature having identified factors associated with sedentary times | Characteristic | Label / unit | | |
| --- | --- | --- | --- | --- | --- |
|  |  |  |  | | |
|  |  |  | **0** | **1** | **2** |
|  |  |  |  |  |  |
| Traffic safety around school | Mantjes et al. 2012; Stierlin et al. 2015(1, 2) | binary | High | Middle or low | - |
| Number of breaks longer than 15 minutes | Mantjes et al. 2012; Morgan et al. 2016(2, 3) | scale |  | | |
| Rules concerning media use in school | Assumption according to Pate et al. 2011(4) | binary | Always | Sometimes or never | - |
| Extracurricular sports programs | Morton et al. 2016(5) | Scale | Number of sport lessons / day | | |
| Availability of play equipment | Mantjes et al. 2012; Ridgers et al. 2013; Stierlin et al. 2015; Morton et al. 2016(5, 2, 6, 1) | binary | Always | Sometimes or never | - |
| Neighborhood quality | Levin et al. 2014; Maitland et al. 2013; Kaushal et al. 2014(7–9) | ordinal | High | Middle | Low |
| Presence of media devices in bedroom | Kaushal et al. 2014; Salmon et al. 2011; Brindova et al. 2014(10, 7, 11) | binary | No | Yes | - |
| Rules concerning media at home | Pate et al. 2011(4) | binary | Yes | No | - |
| Media consumption of parents | Salmon et al. 2011(11) | Scale | scorepoints | | |
| Encouragement for non-sedentary activities | Bauer et al. 2008; Maitland et al. 2013; Brindova et al. 2014(12, 10, 8) | binary | More than sometimes | Sometimes or never | - |

Table S2: Overview on levels of variables

| **Level** | **Variables** |
| --- | --- |
| **Level I: repeated measurements** | - Sedentary behavior (4 outcome variables) - Minutes of MVPA - Number of breaks longer than 15 minutes - Participation at extracurricular sports programs in schools |
| **Level II: Individual data** | - Sex, Age, SES - Traffic safety around school - Rules concerning media use during breaks - Unrestricted availability of play equipment - Neighborhood quality - Presence of media devices in bedroom - Rules concerning media use at home - Media consumption of parents - Encouragement for non-sedentary activities |

Literature Cited

1. Stierlin AS, Lepeleere S de, Cardon G, Dargent-Molina P, Hoffmann B, Murphy MH et al. A systematic review of determinants of sedentary behaviour in youth: a DEDIPAC-study. Int J Behav Nutr Phys Act 2015; 12:133.

2. Mantjes JA, Jones AP, Corder K, Jones NR, Harrison F, Griffin SJ et al. School related factors and 1yr change in physical activity amongst 9-11 year old English schoolchildren. Int J Behav Nutr Phys Act 2012; 9:153.

3. Morgan K, Hallingberg B, Littlecott H, Murphy S, Fletcher A, Roberts C et al. Predictors of physical activity and sedentary behaviours among 11-16 year olds: Multilevel analysis of the 2013 Health Behaviour in School-aged Children (HBSC) study in Wales. BMC Public Health 2016; 16:569.

4. Pate RR, Mitchell JA, Byun W, Dowda M. Sedentary behaviour in youth. Br J Sports Med 2011; 45(11):906–13.

5. Morton KL, Atkin AJ, Corder K, Suhrcke M, van Sluijs EMF. The school environment and adolescent physical activity and sedentary behaviour: a mixed-studies systematic review. Obes Rev 2016; 17(2):142–58.

6. Ridgers ND, Timperio A, Crawford D, Salmon J. What factors are associated with adolescents' school break time physical activity and sedentary time? PLoS One 2013; 8(2):e56838.

7. Kaushal N, Rhodes RE. The home physical environment and its relationship with physical activity and sedentary behavior: a systematic review. Prev Med 2014; 67:221–37.

8. Maitland C, Stratton G, Foster S, Braham R, Rosenberg M. A place for play? The influence of the home physical environment on children's physical activity and sedentary behaviour. Int J Behav Nutr Phys Act 2013; 10:99.

9. Levin KA, Walsh D, McCartney G. OP46 Sedentary behaviour of adolescents in Glasgow compared with the rest of Scotland: The mediating effect of the neighbourhood context. J Epidemiol Community Health 2014; 68(Suppl 1):A24.3-A25.

10. Brindova D, Pavelka J, Sevcikova A, Zezula I, van Dijk JP, Reijneveld SA et al. How parents can affect excessive spending of time on screen-based activities. BMC Public Health 2014; 14:1261.

11. Salmon J, Tremblay MS, Marshall SJ, Hume C. Health risks, correlates, and interventions to reduce sedentary behavior in young people. Am J Prev Med 2011; 41(2):197–206.

12. Bauer KW, Nelson MC, Boutelle KN, Neumark-Sztainer D. Parental influences on adolescents' physical activity and sedentary behavior: longitudinal findings from Project EAT-II. Int J Behav Nutr Phys Act 2008; 5:12.
